# *CASP3* gene expression and the role of caspase 3 in the pathogenesis of depressive disorders

**DOI:** 10.1186/s12888-023-05153-5

**Published:** 2023-09-06

**Authors:** Katarzyna Bliźniewska-Kowalska, Piotr Gałecki, Janusz Szemraj, Kuan-Pin Su, Jane Pei-Chen Chang, Małgorzata Gałecka

**Affiliations:** 1 Department of Adult Psychiatry, Medical University of Lodz, Lodz, Poland; 2 Department of Medical Biochemistry, Medical University of Lodz, Lodz, Poland; 3 Mind-Body Interface Laboratory (MBI-Lab), Department of Psychiatry, China Medical University Hospital, Taichung, Taiwan; 4 College of Medicine, China Medical University, Taichung, Taiwan; 5 Graduate Institute of Biomedical Sciences, China Medical University, Taichung, Taiwan; 6 An-Nan Hospital, China Medical University, Tainan, Taiwan; 7 Department of Psychotherapy, Medical University of Lodz, Lodz, Poland

**Keywords:** *CASP3*, Caspase 3, Depression, Gene expression, Major depressive disorder

## Abstract

**Background:**

The aim of our study was to evaluate the expression of the *CASP3* gene at both mRNA and protein levels in patients with depressive disorders and to determine the impact of caspase 3 in the pathogenesis of depression;

**Methods:**

A total of 290 subjects, including 190 depressed patients and 100 healthy controls, participated in the study. Socio-demographic and clinical data were collected, and the severity of depressive symptoms was assessed using the Hamilton Depression Rating Scale. Venous blood was collected and gene expression was evaluated using RT-PCR and ELISA at the mRNA and protein levels, respectively;

**Results:**

The expression of the *CASP3* gene was significantly lower in depressed patients compared to healthy controls at both the mRNA and protein levels. Additionally, a positive correlation was observed between *CASP3* gene expression and disease duration as well as the number of depressive episodes;

**Conclusions:**

Further studies are needed to investigate the role of caspase 3 in depressive disorders.

## Introduction

Depression is considered a scourge of the modern world. According to the reports of World Health Organization around 5% of adults worldwide experience depression [1]. Despite its high prevalence, the underling pathomechanism of depression is not fully understood. Hence, further studies searching for potential factors contributing to the development of this mental disorder are necessary.

The aim of this study was to evaluate the expression of the *CASP3* gene at the mRNA and protein levels in patients with depressive disorders and to determine the impact of caspase 3 in the etiopathogenesis of depression.

Caspase 3 (encoded by *CASP3* gene) is a major member of the caspase-family of cysteine proteases. It is considered to be a key mediator of apoptosis in neuronal cells. Studies conducted on animal models also suggest that caspase 3 also functions as a regulatory molecule in neurogenesis and synaptic activity [2–4]. Considering the importance of these processes in various mental disorders, including depression, the assessment of the role of caspase 3 in the etiopathogenesis of depressive disorders seems to be justified.

## Materials and methods

A total of 290 subjects(183 F, 107 M) aged 18 to 67 (41.29 ± 13.50 years) participated in the study. The study group consisted of 190 hospitalized patients (117 F, 73 M, mean age: 47.51 ± 11.18 yrs.) with depressive disorders, including both recurrent depressive disorders (F33 according to ICD-10 diagnostic criteria) and a depressive episode (F32 according to ICD-10) [5]. The material for the study was collected from patients in the current depressive episode. Participation in the study did not affect the type of administered treatment. Exclusion criteria from the study included serious mental illnesses other than depressive disorders, serious neurological and somatic diseases, in particular autoimmune and neurodegenerative diseases, active cancer, addiction to alcohol or other psychoactive substances. The control group consisted of 100 healthy individuals (66 F, 34 M, mean age: 29.36 ± 8.71 yrs.) with negative history for psychiatric disorders.

Women predominated in both groups. There was no statistically significant difference between the groups in terms of gender (p = 0.4583). Patients from the study group were statistically significantly older than healthy controls (p < 0.0001 for the multifactor generalized linear model fitted, p < 0.001 for “by-group” comparison, p = 0.528 for “by-gender” comparison), which results from the characteristics of depressive disorders and may constitute a certain limitation of this study. However, the study did assess the correlation between the age of the subjects and other variables (see Results).

**Fig. 1:**
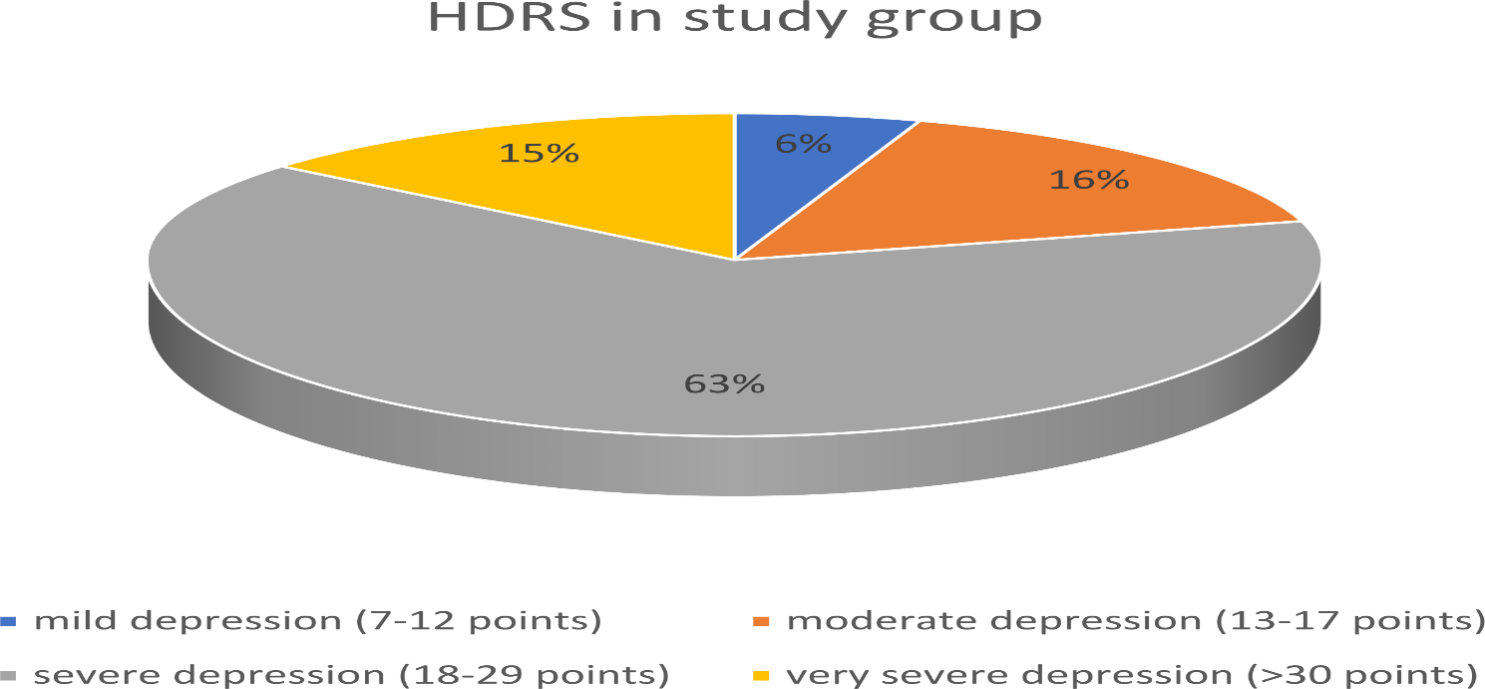
Hamilton Depression Rating Scale (HDRS) results in study group patients

Clinical data on the duration of the disease (in years), the number of depressive episodes, and the number of hospitalizations (including the current one) were collected from the patients from the study group (Table 1). In patients from the study group, the severity of depressive symptoms was also assessed according to the 17-item Hamilton Depression Rating Scale (HDRS) (Fig. 1) [6]. Due to the fact that the study group consisted of hospitalized patients, most of them had severe depressive symptoms (Fig. 1).

**Table 1:** Clinical characteristics of the study group

Analysed trait	Statistical parameter *						
	*M*	*Me*	*Q*_*1*_ *-Q*_*3*_ *(IQR)*	*SD*	*SE*	*95% CI*	*Min.-Max.*
**Number ** **of hospitalizations**	2.01	1	1–2 (1)	2.00	0.15	1.72–2.30	0–12
**Disease duration time (years)**	6.18	4	1–8 (7)	7.05	0.52	5.16–7.20	1–40
**Number ** **of depressive episodes**	4.56	2	1–5 (4)	5.33	0.39	3.79–5.34	1–20

(* Explanations of abbreviations used in result tables: M – mean; Me – median; Q – quartile; SD – standard deviation; SE – standard error; CI – confidence interval)

Venous blood was collected from all study participants (study group and control group) on the day of inclusion in the study for further biochemical analysis. RT-PCR was used to evaluate gene expression at the mRNA level, while enzyme-linked immunosorbent assay (ELISA) was used to evaluate gene expression at the protein level.

Total RNA isolation from the patients’ blood samples (leukocyte monolayer cells) using a RNA extraction reagent, TRIZOL (Invitrogen Life Technologies, Carlsbad, CA, USA), according to the standard acid-guanidinium-phenol-chlorophorm method was performed using modified Chomczyński metod [7].

The quality of total RNA was checked with Agilent RNA 6000 Nano Kit (Agilent Technologies) in accordance with the manufacturer’s recommendations. The quality of isolated RNA was checked using 2100 Bioanalyzer (Agilent Technologies). The level of degradation of total RNA was determined with the use of an electrophoretogram and RIN values recorded. Only the samples with RIN value > 7 were subject to further analysis [8].

An RT reaction was carried out using TaqMan® RNA Reverse Transcription Kit (Applied Biosystems) based on the manufacturer’s recommendations, using specific Hs 00234387_m1, Hs04194366_g1 probes, specific respectively for *CASP3* and RPL13A genes, delivered by Applied Biosystems [8].

Real-Time PCR reaction was conducted using TaqMan® Universal PCR Master Mix, No UNG (Applied Biosystems) according to the protocol provided by the manufacturer. To calculate relative expression of miRNA genes, the Ct comparative method was used [9, 10]. The level of *CASP3* gene expression in blood was normalized in relation to RPL13A reference gene [8].

The concentration of protein caspase 3 in the serum of the patients was determined using Human Caspase Elisa Kit from ThermoFischer Scentific (Waltham, MA, USA)according to the protocols provided by the manufacturer. β-actin was used for endogenous control of protein concentration in the samples and determined with the help of Human Actin Beta (ACTb) ELISA Kit (BMASSAY) based on the manufacturer’s recommendations [8].

The statistical analysis was carried out by using IBM SPSS Statistics, v. 28 (IBM Corporation, Armonk, NY, USA). A level of p < 0.05 was deemed statistically significant. All statistical procedures were set as two-tailed. For contingency cross-tables a chi-squared test was used. Generalized linear models with robust standard errors were performed to test differences in numerical traits between the studied groups. All the models were controlled for the participants’ age and gender. The gene (mRNA) expression levels had been log transformed before testing the hypothesis. There were computed the Pearson correlation coefficient for variables measured on the same scale, and Spearman’s rank correlation coefficient for traits measured on various scales.

## Results

**Fig. 2:**
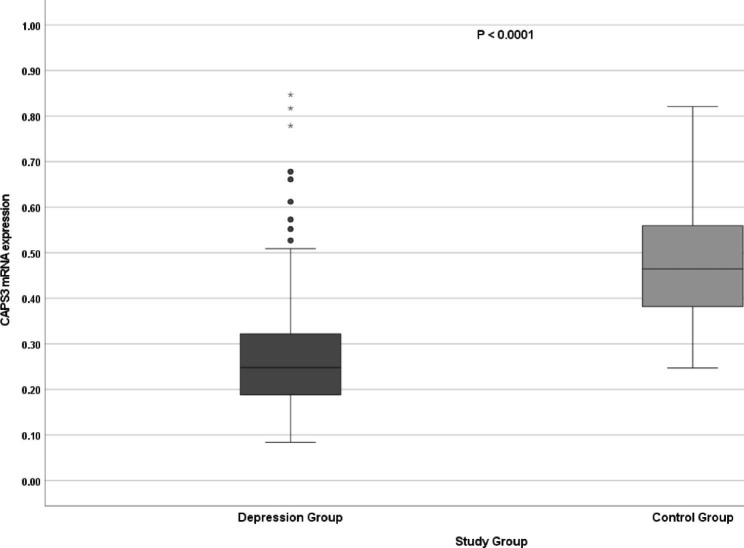
CASP3 gene mRNA expression

**Fig. 3:**
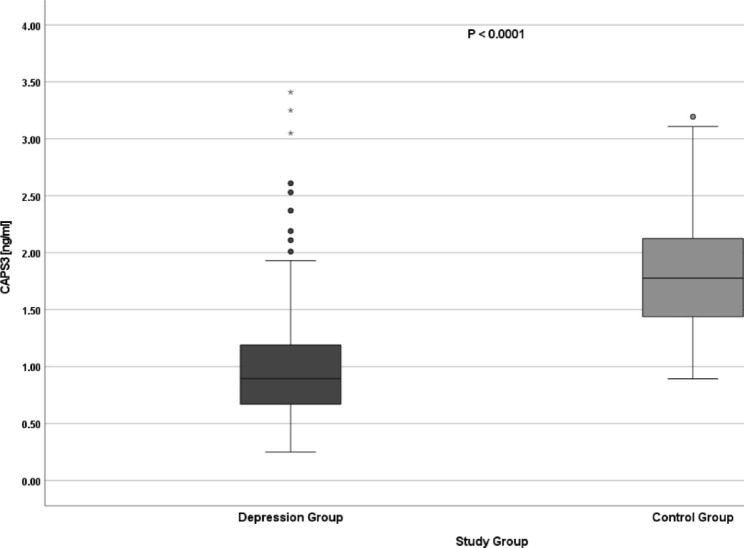
CASP3 gene expression at the protein level

The expression of the *CASP3* gene, both at the mRNA and protein level, was statistically significantly lower in the group of patients with depression than in healthy subjects (Table 2; Figs. 2 and 3).

**Table 2:** Detailed descriptive statistics for *CAPS3* gene expression by study group

Variable	GROUP	Statistical parameter							
		*M*	*Me*	*Q*_*1*_ *-Q*_*3*_ *(IQR)*	*SD*	*SE*	*95% CI*	*Min.-Max.*	*p*
***CAPS3*** **mRNA**	**Study group**	0.276	0.248	0.187–0.323 (0.135)	0.127	0.009	0.258–0.294	0.084–0.847	*p < 0.0001*
	**Control**	0.486	0.464	0.380–0.560 (0.180)	0.135	0.013	0.459–0.513	0.247–0.821	
	**Overall**	0.348	0.313	0.226–0.457 (0.231)	0.164	0.010	0.329–0.367	0.084–0.847	
***CAPS3*** **[ng/ml]**	**Study group**	1.012	0.959	0.670–1.195 (0.525)	0.508	0.037	0.939–1.085	0.250–3.410	*p < 0.0001*
	**Control**	1.851	1.777	1.433–2.129 (0.696)	0.539	0.054	1.744–1.958	0.892–3.194	
	**Overall**	1.301	1.149	0.825–1.719 (0.894)	0.654	0.038	1.226–1.377	0.250–3.410	

*Explanations of abbreviations used in result table: M – mean; Me – median; Q – quartile; SD – standard deviation; SE – standard error; CI – confidence interval; p- statistical significance of differences by study groups*

*All empirical data, considering the gene expression, had been log transformed before testing the hypotheses. All the models fitted were controlled for age and gender.)*

The relationship between the expressions of the studied gene and clinical variables was also determined (Table 3). There was a positive correlation between the disease duration and the *CASP3* gene expression as well as between the number of depressive episodes and the *CASP3* gene expression (Table 3).

**Table 3:** The relationship between the expressions of CASP3 gene and clinical variables

			OVERALL		DEPRESSION		CONTROL	
			*CAPS3* [ng/ml]	*CAPS3* mRNA	*CAPS3* [ng/ml]	*CAPS3* mRNA	*CAPS3* [ng/ml]	*CAPS3* mRNA
Spearman’s rho	Age (years)	Correlation Coefficient	**-0.357** ^**^	**-0.356** ^**^	0.059	0.061	0.142	0.142
		Sig. (2-tailed)	**0.0000**	**0.0000**	0.4224	0.4067	0.1604	0.1612
		N	**289**	**289**	190	190	99	99
	Number of hospitalizations	Correlation Coefficient	0.107	0.102	0.107	0.102		
		Sig. (2-tailed)	0.1471	0.1641	0.1471	0.1641		
		N	186	186	186	186		
	Disease duration (years)	Correlation Coefficient	**0.252** ^**^	**0.237** ^**^	**0.252** ^**^	**0.237** ^**^		
		Sig. (2-tailed)	**0.0005**	**0.0011**	**0.0005**	**0.0011**		
		N	**186**	**186**	**186**	**186**		
	Number of episodes	Correlation Coefficient	**0.236** ^**^	**0.214** ^**^	**0.236** ^**^	**0.214** ^**^		
		Sig. (2-tailed)	**0.0012**	**0.0033**	**0.0012**	**0.0033**		
		N	**186**	**186**	**186**	**186**		
	HDRS	Correlation Coefficient	0.085	0.082	0.085	0.082		
		Sig. (2-tailed)	0.2501	0.2679	0.2501	0.2679		
		N	184	184	184	184		

*Explanations of abbreviations used in result table: Sig. (2-tailed).- the two-tailed p-value, significance, N- number, HDRS- Hamilton Depression Rating Scale*

## Discussion

The name “caspases” comes from the English words “**c**ysteine-dependent, **aspa**rtate-specific peptid**ases**”. They are enzymes from the group of cysteine ​​proteases, which, when activated by apoptosis signals, degrade cellular proteins, cutting the peptide bond behind the aspartate residue. They provide critical links in cell regulatory networks controlling inflammation and cell death [11, 12].

Caspase 3 is an effector caspase, which is activated by caspase 9 (initiator caspase) and plays an important role in apoptosis. Both the extrinsic (death receptor dependent) and intrinsic (mitochrondrial) apoptotic pathways meet at caspase 3. This enzyme is said to be a key mediator of neuronal apoptosis [2]. However, recent studies also indicate important nonapoptotic functions of this enzyme.

Caspase 3 is also involved in the neural differentiation and its activity in neuronal progenitors facilitates neurogenesis [13]. The experiment performed on the clonally derived neurospheres from the striatum of murine embryos showed significant increase in caspase 3 activity during neurosphere differentiation, without the cleavage of PARP (which normally occur during apoptosis), suggesting nonapoptotic role of this caspase in neurogenesis [13]. Additionally the inhibition of caspase 3 activity alters the expression of proteins associated with neurosphere differentiation, like for example nestin or *β*-III tubulin [13]. Moreover, many proteins that are crucial in synaptic plasticity are substrates for caspase 3 [14], which supports the important role of this enzyme in neuroplasticity. The effects of caspase 3 inhibition (via administration of DEVD-fmk) on learning and memory were also evaluated. The intracerebroventricular administration of z-DEVD-fmk decreased the number of avoidance reactions in active avoidance learning in rats. Application of caspase 3 inhibitor to the cerebellar vermis stimulated the extinction of an acoustic startle reaction [2, 15]. Activated caspase 3 is present, in vivo, in the postsynaptic terminal of neurons in the auditory forebrain of small passerine bird of central Australia and is necessary for the development of long-term habituation to a song [16]. If caspase 3 activity contributes to synaptic plasticity, then a mechanism must also exist for limiting its proteolytic effect at the synapse level avoiding the dismantling of the rest of the neuron. For example, the synaptic caspase 3 activity can be suppressed by nine-amino acid active fragment of activity-dependent neurotrophic factor (ADNF-9) [17]. Caspase 3 can be considered a regulatory molecule in neurogenesis and neuroplasticity.

Understanding of the nonapoptotic function of caspase 3 may have potential implications for the comprehension of pathomechanisms of psychoneurological disorders. Numerous studies have demonstrated the neuroprotective effect of the caspase 3 inhibitor Z-DEVD-FMK on rodent models with traumatic brain injury (TBI) [18]. The caspase 3 inhibitor from Merck Frost Canada, L-826791, was revealed to reduce apoptosis in the hippocampus and piriform cortex in preclinical trials for the treatment of brain injury [19, 20]. Application of caspase 3 inhibitors has also proven efficacy in rescuing the Alzheimer-like phenotypes in mice models [21]. Other studies on AD-animal models indicate that caspase inhibitors might prevent cleavage of tau protein [22], alleviate cognitive impairment and delay cognitive decline [20, 23, 24]. Degeneration of dopaminergic neurons in subjects with Parkinson’s disease by apoptosis has been suggested. Also in case of this neurodegenerative disorder usage of caspase inhibitors lead to significant reduction of dopamine depletion in the striatum and inhibited the loss of dopaminergic neurons in the substantia nigra [20, 25, 26]. The treatment with caspase inhibitors (including caspase 3 inhibitors) was able to provide neuroprotective effects in a rodent model with Huntington’s disease [20, 25, 27]. However, it is worth mentioning that the application of caspase inhibitors is limited to preclinical studies on animal models.

Depressive disorders and neurodegenerative diseases are clinically recognized as two entirely different entities; however, they can often cooccur. The prevalence of depression can be as high as 90% in Alzheimer’s disease and 50% in patients with Parkinson’s disease [28]. This common concomitant presentation of these disorders raises many questions regarding the pathophysiological links between these disorders. There are some overlapping pathomechanism, that are present both in depression and neurodegenerative diseases like neuroinflammation [29] and the disturbances in monoamine neurotransmission [30], hypothalamus-pituitary-axis dysfunction, decreased levels of brain-derived neurotrophic factor (BDNF) and increased oxidative stress levels [29, 31].

Depression leads to neuroplasticity changes in specific regions of the brain which are correlated to symptom severity, negative emotional rumination as well as fear learning. Depression is correlated with atrophy of neurons in the cortical and limbic brain regions that control mood and emotion [32–34].

Considering the importance of neurogenesis and neuroplasticity processes in the etiopathogenesis of depression, we decided to investigate the expression of the gene for caspase 3 in patients hospitalized for depressive disorders and in healthy volunteers. In our study the expression of the *CASP3* gene, both at the mRNA and protein level, was statistically significantly lower in the group of patients with depression than in healthy controls. We are not aware of any other studies on the expression of the *CASP3* gene in this group of patients. In contrast, *Szymona et al.*. (2019) reported significantly higher expression of *CASP3* gene in schizophrenic patients compared to the controls [35]. *CASP3* protein was also up-regulated in fibroblasts of patients with Down syndrome [36]. Our study may suggest that apoptotic and neuroplasticity processes are not as important as previously suggested in the etiopathogenesis of depression. However, it is important to remember that expression does not necessarily translate into enzyme activity and the samples in our study were collected from the peripheral blood, not as it is possible on animal models from the neuronal tissue.

## Limitations

Studies indicate that determinations from peripheral blood of expression at the mRNA level and at the protein level for genes largely reflect expression in the central nervous system [37], but there is a lack of comparison in the available literature of results for CASP3 gene in patients with depressive disorders.

Hence, the study was conducted on hospitalized psychiatric patients most of them already received treatment before admission. In order to minimalize the effect of treatment on gene expression, the blood was collected at the beginning of the hospitalization, when the depressive symptoms were the most severe and before the modification of existing antidepressant treatment. Although, an effect of treatment on the expression of the studied genes cannot be ruled out.

## Conclusions

Despite their high prevalence, the etiopathogenesis of depressive disorders is not fully understood. Seeking for novel biomarkers, contributing factors and possible therapeutic targets in depression is necessary. Numerous preclinical animal model studies provide a lead for further investigations in understanding the exact roles of caspase 3. Our study suggest that *CASP3* might play a role in pathogenesis of depression. However further studies are needed to understand the exact role of this enzyme in depression and to provide a better approach for targeting caspases and therapeutic advantage.

## Data Availability

The data that support the findings of this study are available upon reasonable request from author M.G.
